# HAX-1 Protects Glioblastoma Cells from Apoptosis through the Akt1 Pathway

**DOI:** 10.3389/fncel.2017.00420

**Published:** 2017-12-21

**Authors:** Xin Deng, Laijun Song, Wen Zhao, Ying Wei, Xin-bin Guo

**Affiliations:** ^1^Department of Neurosurgery, The First Affiliated Hospital of Zhengzhou University, Zhengzhou, China; ^2^Key Laboratory of Advanced Pharmaceutical Technology, Ministry of Education of China, Zhengzhou, China; ^3^Co-innovation Center of Henan Province for New Drug R & D and Preclinical Safety, Zhengzhou, China; ^4^School of Pharmaceutical Sciences, Zhengzhou University, Zhengzhou, China; ^5^Department of Neuro-interventional Radiology, The First Affiliated Hospital of Zhengzhou University, Zhengzhou, China

**Keywords:** glioblastoma, HAX-1, Akt1, Hsp90, apoptosis

## Abstract

Glioblastoma is the most common malignant tumor in central nervous system (CNS), and it is still insurmountable and has a poor prognosis. The proliferation and survival mechanism of glioma cells needs to be explored further for the development of glioma treatment. Hematopoietic-substrate-1 associated protein X-1 (HAX-1) has been reported as an anti-apoptosis protein that plays an important role in several malignant tumors. However, the effect and mechanism of HAX-1 in glioblastomas remains unknown. This study aimed to investigate the effect of HAX-1 in glioblastoma cells and explore the mechanism. The results of clone formation and Edu proliferation assay showed slower multiplication in HAX-1 knock-out cells. Flow cytometry showed cell cycle arrest mainly in G0/G1 phase. Apoptosis due to oxidative stress was increased after HAX-1 was knocked out. Western-blot assay exhibited that the levels of p21, Bax, and p53 proteins were significantly raised, and that the activation of the caspase cascade was enhanced in the absence of HAX-1. The degradation rate and ubiquitination of p53 declined because of the decrease in phosphorylation of proteins MDM2 and Akt1. Co-immunoprecipitation (Co-IP) and immunefluorescent co-localization assays were performed to test the influence of HAX-1 on the interaction between Akt1 and Hsp90, which is crucial for the activity of Akt1. In conclusion, this novel study suggested that HAX-1 could affect the Akt1 pathway through Hsp90. The knock-out of HAX-1 leads to the inactivity of the Ak1t/MDM2 axis, which leads to increased levels of p53, and finally generates cell cycle arrest and results in the apoptosis of glioblastoma cells.

## Introduction

Glioblastoma is the most common and invasive tumor in the central nervous system (CNS). It is a highly aggressive, rapidly growing malignant neoplasm (Ostrom et al., 2015; Codrici et al., 2016; Maugeri et al., 2016). Relative to its rapid growth rate, its blood supply can become insufficient, causing the central part of the tumor to often suffer from ischemic necrosis. Regardless, glioblastoma cells can still proliferate rapidly in hypoxic conditions, which means some cytoprotective mechanisms exist. Studying these mechanisms may provide us novel ideas for glioblastoma therapy and treatment.

In our previous study, hematopoietic-substrate-1 associated protein X-1 (HAX-1), an anti-apoptotic protein, was found to be overexpressed in glioblastoma cells and tissues (Deng et al., 2017), which has also been reported in other malignant tumors (Trebinska et al., 2010; Luo et al., 2011; Li et al., 2013, 2015; Gomathinayagam et al., 2014; Wei et al., 2014; You et al., 2015, 2016). HAX-1 can inhibit apoptosis by multiple pathways, so HAX-1 may have an important role in the survival of glioblastoma cells in hypoxic-ischemic environments, which still remains unclear. Tumor suppressor p53 has many mechanisms of anti-cancer function by inducing cell cycle arrest and apoptosis (Vaseva and Moll, 2009). Activated p53 binds to deoxyribonucleic acid (DNA) and increases the expression of p21, which interacts with cyclin-dependent kinase (CDK), an protein important for G1/S transition in the cell cycle, to hold the cell cycle at the G1/S regulation point (Barr et al., 2017). P53 can also interact and upregulate the expression of Bcl-2-associated X protein (Bax). This enhances the release of cytochrome c from mitochondria, activating caspase-9 to induce apoptosis by opening the mitochondrial voltage-dependent anion channel (VDAC) (Westphal et al., 2013). As described before, HAX-1 could affect mitochondrial apoptosis, but the relationship between HAX-1 and p53, a crucial regular of mitochondrial apoptosis, is still unknown. HAX-1 can interact with heat shock protein 90 (Hsp90), a chaperone protein. Hsp90 assists other proteins to fold properly and stabilizes proteins from degradation (Prodromou and Pearl, 2003). By interacting with Hsp90 (Lam et al., 2013), HAX-1 promotes the degradation of cyclophilin-D (Cyp-D) to enhance apoptosis (Lam et al., 2015). Interestingly, Hsp90 is also involved in the regulation of the AKT serine/threonine kinase 1 (Akt1) signaling pathway (Sato et al., 2000; Giulino-Roth et al., 2017), which regulates the activation and degradation of p53 protein by controlling the phosphorylation of MDM2 (Wang et al., 2017).

Therefore, we hypothesize HAX-1 might influence apoptosis by regulating p53 protein through the interaction of Hsp90 and Akt1. The current study was designed to test this hypothesis by altering the expression of HAX-1 in glioblastoma cell lines U118 and U87-MG. Our findings indicate that HAX-1 could increase the phosphorylation level of Akt1 by enhancing the combination of Akt1 and Hsp90. Down-regulation of HAX-1 decreased activity of Akt1 and MDM2, subsequently causing a decline in ubiquitination and degradation of p53, which lead to cell cycle arrest and apoptosis via increased expression of p21 and Bax. Taken together, our results reveal a novel mechanism by which the HAX-1/Hsp90 complex may enhance the activation of the Akt1/MDM2/p53 axis, and promote U118 and U87-MG survival under oxidative stress.

## Materials and Methods

### Cell Cultures

Human glioblastoma cell lines U87-MG and U118 were purchased from the Cell Bank of the Chinese Academy of Sciences, cultured in dulbecco’s modified eagle medium (DMEM)/HIGH GLUCOSE medium (HyClone, United States) with 10% fetal bovine serum (FBS, Gibco), 0.5 mm glutamine, and 100 U/mL penicillin/streptomycin at 37°C in humidified atmosphere containing 5% CO_2_ and 95% air.

Human astrocytes (HA) were purchased from ATCC, cultured in DMEM/HIGH GLUCOSE medium (HyClone, United States) with 10% fetal bovine serum (FBS, Gibco), 0.5 mm glutamine and 100 U/mL penicillin/streptomycin at 37°C in humidified atmosphere containing 5% CO_2_ and 95% air.

### Viral Infection

In order to knock out HAX-1 protein expression in glioblastoma cells, we delivered a Lentivirus with CRISPR/Cas9 sequence and two kinds of single-guide ribonucleic acid (sgRNA) sequence. The negative control virus carried green fluorescent protein GFP only. All viruses were constructed and packed by Shanghai GeneChem. Lentiviruses were used to infect glioblastoma cells with a multiplicity of infection (MOI) of 10 in the second day of culture. The efficiency of gene transfer was evaluated by the expression of GFP. Nearly 100% of U118 and U87-MG cells appeared infected at 10 MOI at the third day after infection. The sgRNA sequence was designed as follows:

HAX1-sgRNA-1: 5′-CCCCCCAACCAGCACCAGAC-3′,
HAX1-sgRNA-2: 5′-TGCTATTGAAATCTCGTACT-3′

U118 and U87-MG cells were counted and placed in 6 pore cell culture plates, and were all infected with either Adenovirus HAX-1 or Adenovirus GFP control at MOI 500 for 12 h after cells adherence. The efficiency of gene transfer by adenovirus was evaluated by the expression of GFP. Almost 100% cells appeared infected at 500 MOI by 24 to 48 h.

The infected U118 and U87-MG cells were washed with PBS and harvested for quantitative immunoblotting or used in the experiments outlined in the results.

### Colony Formation Assay

U118 and U87-MG cells with different HAX-1 expression were seeded in a 6-well plate (5000 cells per well) and continuously cultured for 7 days. After washing with PBS, cells were fixed with ice-cold methanol and stained with crystal violet solution (Sigma–Aldrich, United States) (0.1% in 25% methanol). The crystal violet crystals were dissolved by 70% ethanol after images were photographed, and the absorbance of supernatant was measured at 595 nm.

### Cell Viability

Both HAX-1 positive and negative glioblastoma cells were seeded into 24-well plates (50000 cells per well) overnight and then incubated with 5-ethynyl-2′-deoxyuridine (Edu) for 2 h. Then cells were washed with PBS and fixed in 4% paraformaldehyde overnight at 4°C. After glycine termination, chromogenic buffer containing kFluor640 was added. 4′,6-diamidino-2-phenylindole (DAPI) stain was performed after the cells were washed twice with PBS. High content screening system (HCS) (Thermo scientific, United States) was used to detect and analyze the positivity of Edu staining.

### Cell Cycle Analysis

Cells were harvested and washed twice with PBS. Then the cells were fixed in 75% ethanol on ice for 30 min. After being washed with PBS twice again, cells were stained using a solution containing 0.2 mg/mL DNase-free RNase A and 10/mL propidium iodide (PI) (KeyGEN BioTECH) for 30 min. Flow cytometry was performed and over 10,000 cells per sample group were collected. Data was analyzed by the Modfit software (BD Biosciences).

### Immunofluorescence

Cells were washed with PBS and fixed in 4% paraformaldehyde over night at 4°C. Immunofluorescent staining was performed by using Opal^TM^ 7-color manual IHC Kits (PerkinElmer, United States). The cells were pretreated with microwave in AR buffer for 15 min and then blocked in block solution for 10 min. The primary antibody was incubated at 37°C for 2 h. After the cells were washed with TBST, secondary antibodies were used for 15 min (HRP conjugated, PerkinElmer, United States). Opal fluorophore working solution was then incubated for 10 min. After microwave treatment in AR buffer, another staining cycle was performed as describe above. Hsp90, Akt1 and HAX-1 were detected by different primary antibody (Hsp90 from Proteintech, China, Akt1 from CST, United States, and HAX-1 from BD, United States) and stained by different Fluorescent dyes (Hsp90 stained with red fluorescence, Akt1 stained with green fluorescence and HAX-1 stained with blue fluorescence). Laser scanning confocal microscopy (LSCM, Nicon) was used for cell observation.

### RNA Extraction, cDNA Synthesis, and Real-time Quantitative PCR

After a week of lentivirus infection, total RNA was extracted from U118 and U87-MG using RNAiso Plus (Takara, Japan), chloroform, and isopropyl alcohol. Then 1 μg of extracted RNA was reverse transcribed into strand cDNA after removing genomic DNA using a PrimeScript RT reagent kit with genomic DNA (gDNA) Eraser (Takara Biotechnology Co., Ltd.). Real-time quantitative polymerase chain reaction was performed on a Roche LightCycler using the same reagent kit with cDNA synthesis for desired genes. The primers were designed and synthesized by Sangon Biotech as follows:

HAX-1: sense: 5′-ACGCCTCGCTCAATTTCTCA-3′;
antisense: 5′-AAGCCAAATTCCTCAGGGGG-3′.

P53: sense: 5′-TGCTCAAGACTGGCGCTAAA-3′;
antisense: 5′-TTTCAGGAAGTAGTTTCCATAGGT-3′;
Glyceraldehyde-3-phosphate dehydrogenase: sense: 5′:
TCCTGCACCACCAACTGCTTAG-3′;
antisense: 5′–TCCTGCACCACCAACTGCTTAG-3′.

### Cell Apoptosis Assay

1ˆ10 (Luo et al., 2011) cells were harvested and washed with PBS. Cells were centrifuged and resuspended in binding buffer contains Annexin-V APC (KeyGEN BioTECH, China) and propidium iodide(PI) (KeyGEN BioTECH, China), which were excited at 633 and 488 nm and emitted fluorescence at 660 and 610 nm, respectively. Flow cytometric assays were performed and over 10,000 cells per sample group were collected. The percent of Annexin-V positive population in glioblastoma cells was calculated as an indication of apoptosis.

### Co-immunoprecipitation

Cells were homogenized in cell lysis buffer (Solarbio), supplemented with 1 mM PMSF and complete protease inhibitor mixture (Solarbio). The homogenate was centrifuged at 11,000 rpm/min for 15 min at 4°C. The supernatant was incubated with mouse-anti-human Hsp90 (Proteintech), rabbit-anti-human Akt1 (CST), p53, or ubiquitin (Proteintech) crosslinked beads at 4°C overnight with rotation. The pretreatment of beads and the immunoprecipitation step were carried out according to the Pierce^TM^ Crosslink Magnetic IP Kit instruction. The identification of the associated proteins was detected by western blot. Homogenates from co-culture cells were used as positive controls.

### Quantitative Immunoblot Analysis

Cells were collected and homogenized in 1× cell lysis buffer (Solarbio) supplemented with 1 mM phenylmethylsulfonyl fluoride, phosphatase inhibitor, and complete protease inhibitor mixture (Solarbio) for 30 min on ice. They were then centrifuged at 11,000 rpm for 10 min at 4°C. The supernatant was assessed using a Bicinchoninic Acid Protein Assay Kit (Solarbio). After measurement, the samples were combined with 5× sodium dodecyl sulfate loading buffer and boiled at 100°C for 10 min. For each protein, equal amounts of samples (20–100 μg) from each group were analyzed by SDS–polyacrylamide gel electrophoresis as previously described. After proteins were transferred onto a polyvinylidene difluoride membrane, the membrane was incubated with 5% BSA at room temperature for 2 h to block the non-specific protein site, and then with corresponding primary antibodies (mouse-anti-human HAX-1 from BD, Hsp90 from Proteintech; Rabbit anti human p21, p53, Bax, ubiquitin from Proteintech, Caspase-3, Caspase-9, PARP, MDM2, pMDM2, Akt1, pAkt1 from CST, glyceraldehyde-3-phosphate dehydrogenase (GAPDH) and β-actin from ZSGF-BIO, China) at 4°C overnight. This step was followed by incubation with HRP-conjugated secondary antibodies (ZSGF-BIO). Visualization was achieved using a SuperSignal West Pico Trial Kit (Thermo).

### Statistical Analysis

Data was expressed as the Mean ± SD. One-way ANOVA analysis of difference was used for comparisons among multiple groups, followed by ordinary one-way ANOVA multiple comparisons. Unpaired student’s *t*-test was performed for comparisons of the means of two groups. Graphpad 6.0 was used for all the statistical analyses. Results were considered statistically significant at *P* < 0.05.

## Results

### HAX-1 Regulated Glioblastoma Cell Proliferation

**FIGURE 1:**
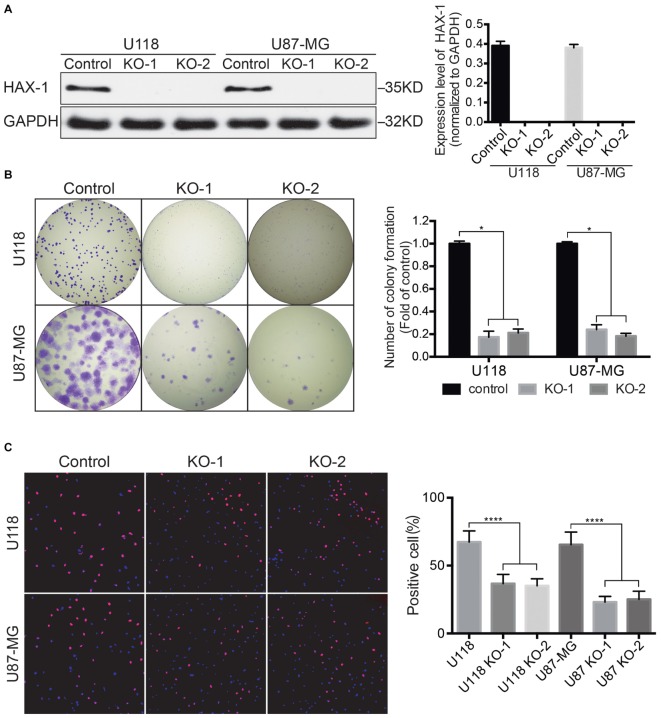
HAX-1 regulated cell proliferation of glioblastoma cells. **(A)** Western blot showed that HAX-1 was completely knocked out in U118 and U87-MG. GAPDH was used as a loading control. **(B)** Colony formation assays indicated that the efficiency of colony formation of U118 and U87-MG cells declined after HAX-1 was knocked out. **(C)** Edu proliferation assays showed decreased proliferative U118 and U87-MG cells. Edu was labeled with red fluorescence and nuclei were stained with blue fluorescence. (magnification: 100×) Three individual experiments were performed for each group. ^∗^*P* < 0.05, ^∗∗∗∗^*P* < 0.0001.

As shown in **Figure 1A**, HAX-1 was knocked-out completely in the KO-1 and KO-2 groups. The efficiency of clone formation of HAX-1 knock-out U118 and U87-MG was markedly reduced compared with control (*p* < 0.05, **Figure 1B**). Edu proliferation assay was used to further determine the effect of HAX-1 knockout. Compared with control group, each of the proliferation rates of the HAX-1 knock out cell lines, KO-1 and KO-2, of both U118 and U87-MG were reduced significantly (*p* < 0.0001, **Figure 1C**). These results show us that HAX-1 knock out causes slower proliferation of U118 and U87-MG cells.

### HAX-1 Knock-out Induced Cell Cycle Arrest in Glioblastoma Cell Lines

**FIGURE 2:**
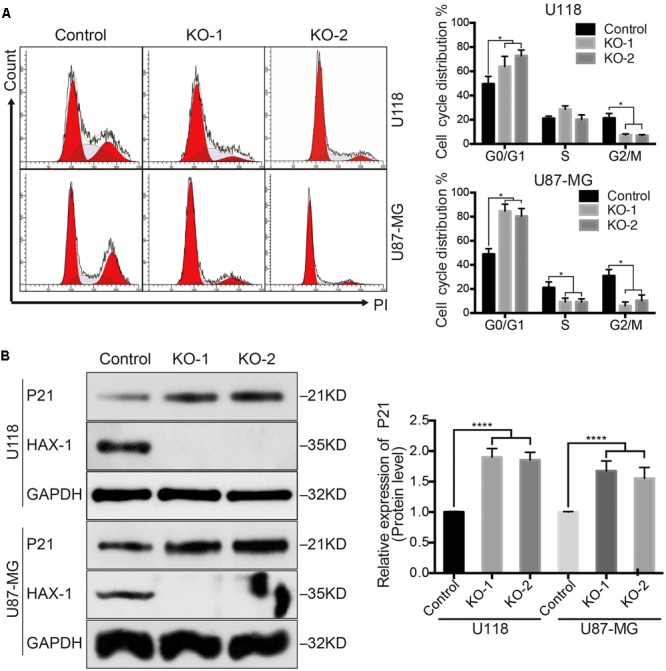
Knock out HAX-1 induced cell cycle arrest in glioblastoma cell lines. **(A)** Cell cycle assays showed in both groups of HAX-1 knock-out cells (U87-MG and U118) that KO-1 cells arrest in G0/G1 phase, and in U118 KO-2 cells mainly arrest in S phase (the first red peak corresponds to the G0/G1 phase, the second red peak corresponds to the G2/M phase, and the gray are area represents S phase). **(B)** Western blot indicated protein p21 was overexpressed after HAX-1 was knocked-out. GAPDH was used as a loading control. Three individual experiments were performed for each group. ^∗∗∗∗^*P* < 0.0001.

To further investigate the effect of HAX-1 on glioblastoma cell proliferation, we examined the cell cycle through flow cytometry. The percentage of cells in G0/G1 phase was increased 1.73- and 1.65-fold in HAX-1 U87-MG cells transfected with KO-1 and KO-2 separately, compared to control group (**Figure 2A**). Similarly, HAX-1 knock out also resulted in G0/G1 and S phase arrest in U118 cells (**Figure 2A**). Canonically, alteration of a G0/G1 checkpoint proteins’ expression, such as p21, is related with G0/G1 arrest. So, we investigated whether HAX-1 could affect the expression of p21. Our results showed that HAX-1 knock-out significantly increased the expression of p21 in both U118 and U87-MG cells (**Figure 2B**). These results indicated that HAX-1 may regulate cell proliferation by influencing p21 expression.

### HAX-1 Knock-out Enhanced Apoptosis of U118 and U87-MG Cells in Oxidative Stress

**FIGURE 3:**
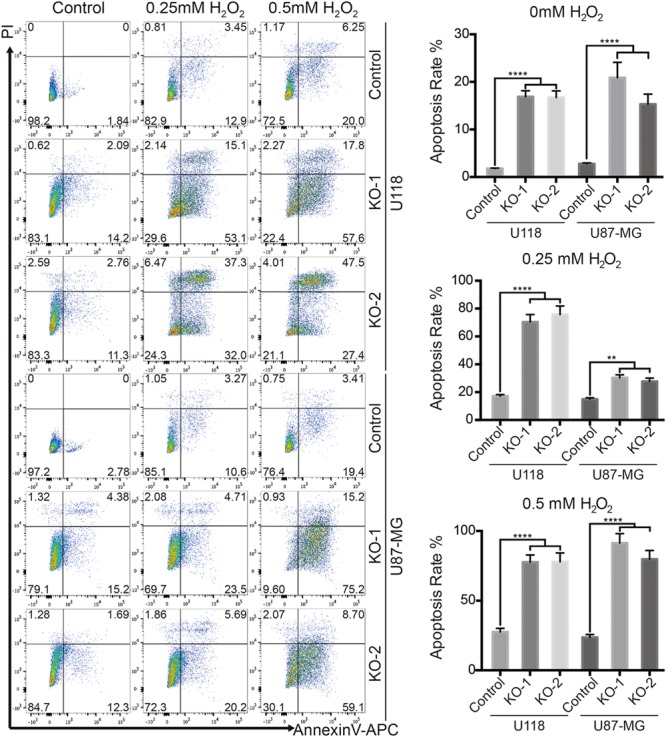
HAX-1 knock-out enhanced apoptosis of U118 and U87-MG cells under oxidative stress. Cell apoptosis assay showed that HAX-1 knock-out obviously increased the apoptosis of U118 and U87-MG cells. Apoptosis rates were measured by FCM analysis after Annexin-APC and PI staining. Three individual experiments were performed for each group. ^∗^*P* < 0.05, ^∗∗^*P* < 0.01, ^∗∗∗^*P* < 0.001, ^∗∗∗∗^*P* < 0.0001.

HAX-1 is a well-known anti-apoptosis protein, so we investigated the effect of HAX-1 on glioblastoma cell apoptosis. Hydrogen peroxide which could release oxyradical to induce oxidative stress was used in this research. As shown in **Figure 3**, HAX-1 knock-out increased the rate of cell apoptosis slightly in both U118 and U87-MG cells. Remarkably, the cell apoptosis rate increased in HAX-1 knock-out U118/U87-MG cells compared to control when cells were pretreated with low doses of hydrogen peroxide. As the dose of hydrogen peroxide increased, the rate of apoptosis of HAX-1 knock-out U118/U87-MG cells increased significantly to over 75%, while apoptosis in control U118/U87–MG cells remained at a lower level, below 30% (**Figure 3**). These results indicated that HAX-1 plays a protected role in glioblastoma cell apoptosis, and HAX-1 knock-out could increase the sensitivity of U118 and U87-MG cells to apoptosis.

### HAX-1 Knock-out Could Increase Caspase Activation

**FIGURE 4:**
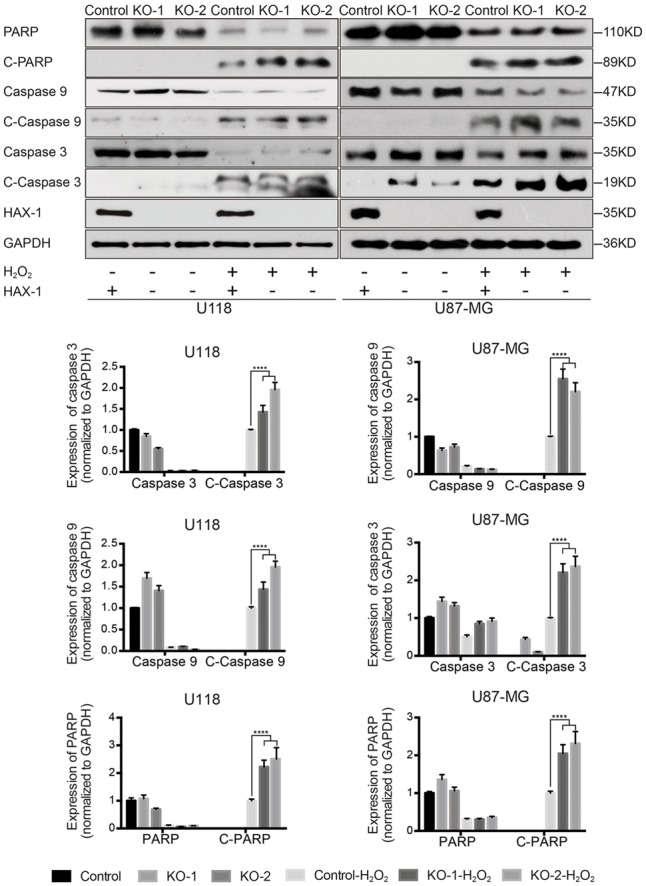
HAX-1 knock-out could increase caspase activation and affect apoptotic protein expression. Western blot indicated the caspase cascade was over-activated in the absence of HAX-1. GAPDH was used as a loading control. Three individual experiments were performed for each group. ^∗∗∗∗^*P* < 0.0001.

**FIGURE 5:**
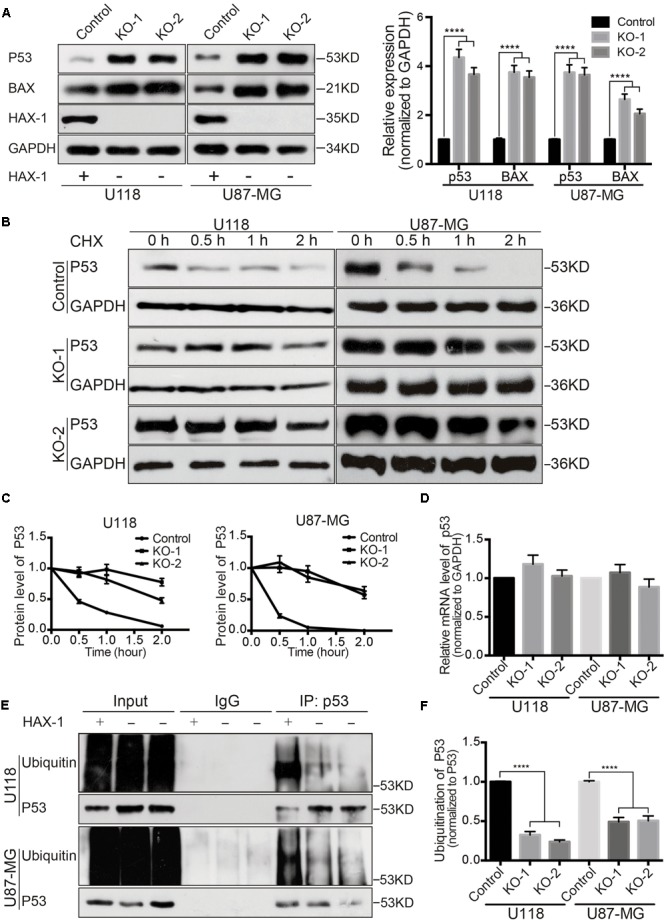
HAX-1 is involved in the regulation of the degradation of p53 protein. **(A)** HAX-1 knock-out enhanced the expression of p53 and Bax. GAPDH was used as a loading control. **(B,C)** Western-blot showed the degradation rate was slowed down in the absence of HAX-1. GAPDH was used as a loading control. **(D)** qRT-PCR indicated the level of p53 mRNA did not alter with the expression of HAX-1. GAPDH was used as a loading control. **(E,F)** The ubiquitination of p53 was reduced in HAX-1 knock-out U118 and U87-MG cells. P53 was used as a loading control. Three individual experiments were performed for each group. ^∗∗∗∗^*P* < 0.0001.

HAX-1 has been reported to regulate apoptosis via the mitochondria and endoplasmic reticulum (Lam et al., 2015; Yan et al., 2015). Our prior results indicated that HAX-1 is involved in apoptosis of U118 and U87-MG cells apoptosis, therefore the changes in apoptotic proteins after HAX-1 knock-out were investigated in subsequent research. Western blot showed the protein levels of cleaved PARP, cleaved Caspase-3, and cleaved Caspase-9 were increased 1.4-, 2.3-, and 1.3-fold respectively in HAX-1 knock-out U118 cells compared to control after hydrogen peroxide treatment. Similarly, HAX-1 knock out also resulted in increase of caspase activation in U87-MG cells (**Figure 4**). Furthermore, we found that the levels of p53 and Bax were upregulated by HAX-1 knock-out in both U118 and U87-MG cells (**Figure 5A**). These results indicated that HAX-1 knock-out enhanced the activation of the caspase cascade and is involved in regulating the of expression of p53 and Bax, which might be a new mechanism for HAX-1 anti-apoptotic effect.

### HAX-1 Is Involved in the Regulation of the Degradation of p53 Protein through Akt1 Pathway

As a tumor-suppressor protein, p53 plays a major role in cellular response to DNA damage. Increase of p53 can lead to either cell cycle arrest or apoptosis. According to our results in a previous study, HAX-1 knock-out in U118 and U87-MG cells induced cell cycle arrest and sensitized the cells to apoptosis, while at the same time the p53 protein level was significantly increased. Therefore, we investigated the relationship between HAX-1 and p53.

Compared with the control group, p53 protein degradation slowed after Cycloheximide (CHX) blocking protein synthesis (**Figures 5B,C**). Using qRT-PCR to detect p53, we found no significant difference between control and HAX-1 knock out group (**Figure 5D**). These findings imply that HAX-1 might regulate the degradation of p53. IP was performed to analyze the ubiquitination level of p53, and showed that HAX-1 knock-out notably decreased the level of ubiquitin (**Figures 5E,F**). All of these results illustrated that HAX-1 might regulate the degradation of p53 by affecting the ubiquitination of p53.

### HAX-1 Enhances the Phosphorylation of Akt1

**FIGURE 6:**
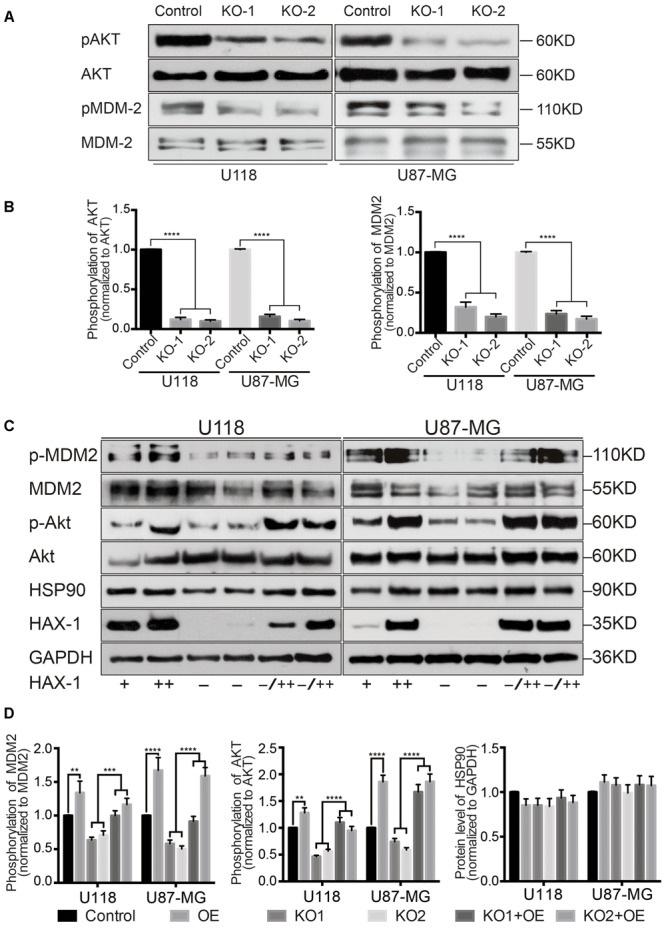
HAX-1 is involved in the phosphorylation of Akt1 and MDM2. **(A,B)** The phosphorylation of MDM2 and Akt1 was investigated, and HAX-1 knock-out reduces the level of phosphorylation. Akt1 and MDM-2 were used as loading controls. **(C,D)** Western blot showed the phosphorylation level of Akt1 and MDM-2 and the expression of Hsp90 with different HAX-1 levels. Akt1, MDM-2 and GAPDH were used as loading controls. Three individual experiments were performed for each group. ^∗∗^*P* < 0.01, ^∗∗∗^*P* < 0.001, ^∗∗∗∗^*P* < 0.0001.

The ubiquitination and proteasomal degradation of p53 is regulated by MDM2, which is regulated by Akt1 protein. Thus, the phosphorylation of MDM2 and Akt1 was investigated, and both phosphorylation of MDM2 and Akt1 was decreased after HAX-1 was knocked out (**Figures 6A,B**). In order to further identify the role of HAX-1 in the phosphorylation of Akt1, we overexpressed HAX-1 by Adenovirus transfection. Compared with control and HAX-1 KO group, HAX-1 expression level was increased in overexpressed (OE) groups. Correspondingly, the phosphorylation level of MDM2 and Akt1 was increased 1.3-, 1.67-, 1.28-, and 1.85-fold in U118 and U87-MG cells separately, while there was a decline in the KO-1 and KO-2 groups, which was reversed by the overexpression of HAX-1 (**Figures 6C,D**). These results indicated HAX-1 could enhance the phosphorylation of Akt1, which lead the increase of phosphorylation of MDM2 and ubiquitination of p53.

### HAX-1 Influences the Phosphorylation of Akt1 through the Interaction with Hsp90

**FIGURE 7:**
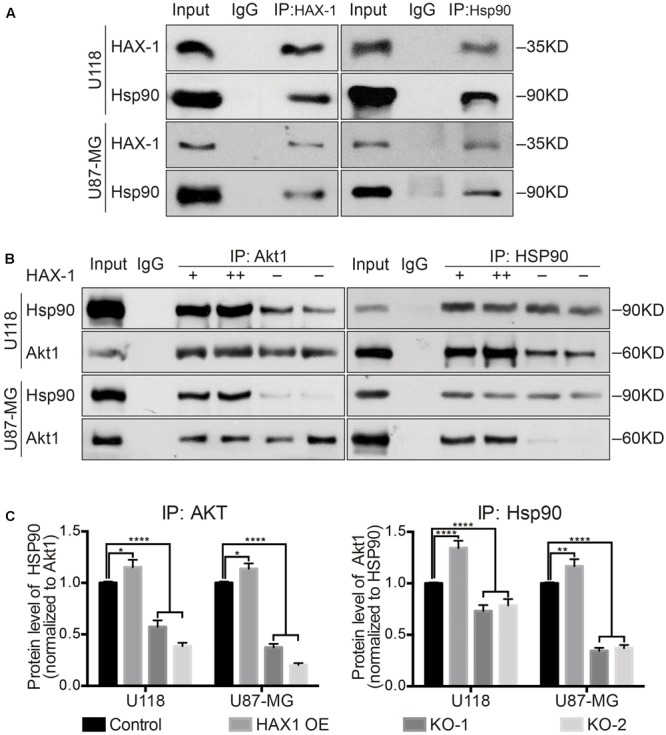
HAX-1 influences the phosphorylation of Akt1 through interaction with Hsp90. **(A)** Co-IP showed HAX-1 combined with Hsp90 in U118 and U87-MG cell lines. **(B,C)** Co-IP exhibited that HAX-1 influenced the interaction between Hsp90 and Akt1. Three individual experiments were performed for each group. ^∗^*P* < 0.05, ^∗∗^*P* < 0.01, ^∗∗∗^*P* < 0.001, ^∗∗∗∗^*P* < 0.0001.

Previous studies have shown that Akt1 interacts with the heat shock protein 90 (Hsp90) (Sato et al., 2000; Esfahani and Cohen, 2016; Giulino-Roth et al., 2017). Interestingly, Hsp90 is a binding partner of HAX-1 (Lam et al., 2013, 2015). So, we investigated the expression of Hsp90 after altering the expression of HAX-1, and as shown in **Figure 6C**, the protein level of Hsp90 had no significant change in U118 and U87-MG cells with altered levels of HAX-1 expression (**Figures 6C,D**). Next, we investigated whether HAX-1 affects the interaction between Akt1 and Hsp90. Co-IP was performed to verify the combination of HAX-1 and Hsp90 and whether HAX-1 affected the interaction between Hsp90 and Akt1. Co-immunoprecipitation showed that, HAX-1 was combined with Hsp90 (**Figure 7A**). Compared with control group, Hsp90 baited by Akt1 increased 1.15- and 1.12- fold in HAX-1 overexpression U118 and U87-MG cells respectively, and decreased 0.57-, 0.38-, 0.37-, and 0.2- folds in KO-1 and KO-2 transfected U118 and U87-MG cells separately. The same trend was observed of the Akt1 baited by Hsp90 (**Figures 7B,C**).

**FIGURE 8:**
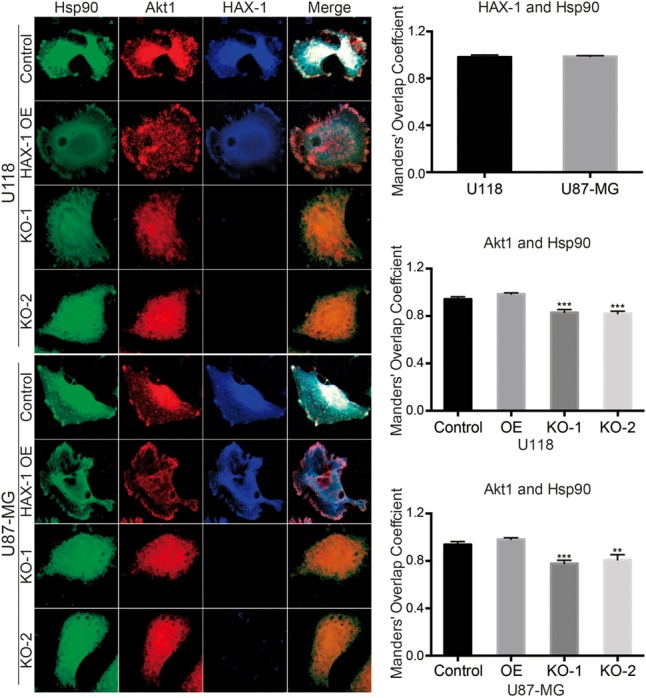
HAX-1 influences the distribution of Akt1 and Hsp90 in U118 and U87-MG cells. Confocal Microscopy showed the co-localization of HAX-1 stained with blue fluorescence, Hsp90 stained with green fluorescence and Akt1 stained with red fluorescence. (original magnification: 600×). Manders’ Overlap Coefficient was chosen as the co-location measurement standard. Three individual experiments were performed for each group. ^∗∗^*P* < 0.01, ^∗∗∗^*P* < 0.001.

Immunofluorescent co-localization was performed to further investigate the effect of HAX-1 on Hsp90 and Akt1. As shown in **Figure 8**, HAX-1 and Hsp90 co-localize in control U118 and U87-MG cells, which supported the combination of HAX-1 and Hsp90. Hsp90 and Akt1 tended to co-localize near the membrane-substrate interface to form a peripheral ring-like structure in control cells. Overexpression of HAX-1 enhanced this trend while knock-out HAX-1 lead the decrease of co-localization between Hsp90 and Akt1, and Hsp90 co-localized with Akt1 in a diffusely distributed punctate pattern throughout the cell (**Figure 8**).

**FIGURE 9:**
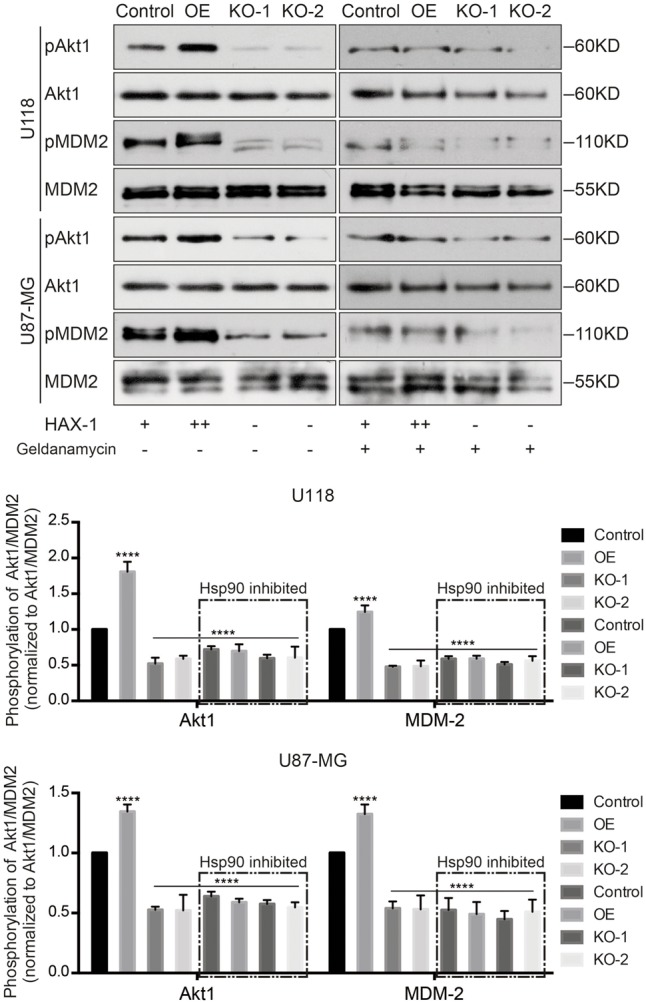
Inhibition of Hsp90 could block the effect of HAX-1 on Akt1. Western blot showed the changes the phosphorylation of Akt1 and MDM-2 induced by HAX-1 alteration were obviously diminished after Hsp90 was inhibited. GAPDH, Akt-1, MDM-2 were used as loading control. Three individual experiments were performed for each group. ^∗∗∗∗^*P* < 0.0001.

To further verify the role of Hsp90, we inhibited Hsp90 by using Selective Hsp90 inhibitor Geldanamycin (APExBIO, United States) in U118 and U87-MG cells. As shown in **Figure 9**, the phosphorylation of MDM2 and Akt1 was significantly decreased after Hsp90 was inhibited, especially in HAX-1 OE group. These results indicated that HAX-1 promoted the binding of Akt1 and Hsp90, which might promote the phosphorylation of Akt1.

## Discussion

In this study, we established HAX-1 knock-out U118 and U87-MG cell lines, and used them to explore the role of HAX-1 in glioblastoma cells. HAX-1 knock-out inhibited U118 and U87-MG cells proliferation and induced cell cycle arrest at G0/G1 and S phase. Protein p21 was significantly increased in HAX-1 knock-out U118 and U87-MG cells. Also, the apoptosis of U118 and U87-MG cells in oxidative stress was increased after HAX-1 knock out, and the caspase cascade was over-activated accordingly. In addition, p53 and Bax were also markedly increased in HAX-1 knock-out glioblastoma cells. We then investigated the degradation of p53 and showed that HAX-1 knock-out notably slowed down the degradation rate of p53 and decreased the ubiquitination levels of p53. Accordingly, the phosphorylation of MDM2 and Akt1 were reduced obviously in HAX-1 knock-out U118 and U87-MG cells. Next we detected the binding of protein Hsp90 and Akt1, and discovered that HAX-1 knock-out reduced the binding of the two proteins, which increased the dephosphorization of Akt1 protein.

HAX-1, as an axiomatic pro-survival protein, was first reported as a causal mutation in congenital neutropenia (Kostmann disease) (Klein et al., 2007). HAX-1 also takes a crucial role in cell protection by depressing the activation of the mitochondrial and ER-related apoptosis pathways (Lam et al., 2013, 2015; Yan et al., 2015). Growing evidence has demonstrated HAX-1 is overexpressed in many kinds of malignant tumors, particularly affecting proliferation, migration, and apoptosis. In our previous work, HAX-1 was found to be overexpressed in glioblastoma tissues and cell lines, and associated with the clinicopathological characteristics and prognosis of glioblastomas (Deng et al., 2017). The role and mechanism of HAX-1 in glioblastoma aroused our interest, so we knocked out the HAX-1 gene in U118 and U87-MG cell lines by CRISPR/Cas9 system, a new convenient and efficient gene compilation technology. Compared with control cells, HAX-1 knock-out U118 and U87-MG cells proliferated at a lower rate and more cells were arrested at G0/G1 and S phase. Further showing how HAX-1 alteration affects the cell cycle, p21 was investigated as a regulator of cell cycle progression at G0/G1 and S phase (Barr et al., 2017). It was found to be increased in the absence of HAX-1. This suggests that HAX-1 could reduce the level of p21 protein, which would promote the end of G0/G1 or S phase and induce U118 and U87-MG cells into the cycle of multiplication. On the other hand, as an anti-apoptotic protein, HAX-1 might affect the apoptosis of U118 and U87-MG cells, a hypothesis our results support. Compared with the HA cells, which expressed HAX-1 at a lower level, less apoptosis occurred in U118 and U87-MG cells with the treatment of hydrogen peroxide. Additionally, the rate of apoptosis increased slightly after the knock-out of HAX-1 in U118 and U87-MG cells, and increased acutely when cells were stimulated with hydrogen peroxide. In addition, the activation of the caspase cascade by hydrogen peroxide was remarkably enhanced when HAX-1 was knocked out. This was particularly demonstrated regarding caspase-9, the marker protein of the mitochondrial apoptosis pathway. These phenomena indicate that HAX-1 influences the proliferation and apoptosis of glioblastoma cells U118 and U87-MG.

In order to explore the mechanism of the pro-apoptotic effect of HAX-1, we detected the protein level of protein p53, which plays a key role in mitochondrial apoptosis (Vaseva and Moll, 2009). The results shows the protein levels of p53 and its downstream protein Bax were notably raised in HAX-1 knock-out U118 and U87-MG cells, which could enhance the release of cytochrome c from mitochondria to activate caspase 9 and thus induce apoptosis by increasing the open of the mitochondrial voltage-dependent anion channel (VDAC) (Westphal et al., 2013). This might explain the over-activation of the caspase cascade in HAX-1 knock-out U118 and U87-MG cells under oxidative stress. P53 can also regulate the cell cycle by increasing protein p21, which is consistent with our previous experiment regarding a cell cycle assay and the detection of p21.

To further explore how HAX-1 regulated p53 expression, we measured the mRNA level of p53. Whether HAX-1 is present or not, there was no significant change in p53 transcription. So we next investigated the degradation rate of p53. Cycloheximide, a classic protein synthesis inhibitor, was applied to detect the degradation of p53. As shown in our results, p53 degraded much more slowly and the ubiquitination of p53 was remarkably decreased after HAX-1 was knocked out. It has been well known that as a negative regulator of p53, MDM2 could bind to p53 to restrict its function and ubiquitinate p53 to promote degradation (Kundu et al., 2017). The activation of MDM2 is regulated by phosphokinase Akt1, which can phosphorylate MDM2 at Ser^186^ to enhance its ubiquitination-promoting function (Wang et al., 2017). Similarly, the activity of Akt1 is also regulated by phosphorylation at Thr^308^ and Ser^473^ (Liu et al., 2014). The phosphorylation level of MDM2 and Akt1 were consistently decreased in the absence of HAX-1, which led to the reduction of p53 ubiquitination. In addition, the phosphorylation level of Akt1 was increased when HAX-1 was overexpressed in U118 and U87-MG cells. This reversal further demonstrated the role of HAX-1 in the regulation of phosphorylation of Akt1. All of these findings indicated that HAX-1 could influence the phosphorylation of Akt1 to regulate the ubiquitination of p53, and therefore regulating the protein level of p53, which affects the cell cycles and apoptotic fates of U118 and U87-MG cells.

In a previous study, HAX-1 was reported to interact with Hsp90 (Lam et al., 2013, 2015), a chaperone protein. Hsp90 assists other proteins to fold properly and can stabilize a number of proteins involved in tumor growth, which makes Hsp90 an investigatory target in cancer research (Esfahani and Cohen, 2016). In particular, HAX-1 has been reported in recent years to combine with Hsp90 to adjust the expression of Cyclophilin-D and IRE-1, which can separately regulate the mitochondrial apoptosis and ER stress-related apoptosis (Lam et al., 2013, 2015). Hsp90 was also reported to be involved in the phosphorylation of Akt1. The activation of Akt1 requires phosphorylation by 3-phosphoinositide-dependent protein kinase-1 (PDK-1) at two key regulatory sites, Thr^308^ and Ser^473^ (Sato et al., 2000; Lim et al., 2017; Zhao et al., 2017). Protein phosphatase 2A (PP2A) can mediate dephosphorylation to inactivate Akt1. Akt1 can bind to the middle domain of Hsp90 to form a complex and phosphorylate the substrate. Hsp90 acts a scaffold during the phosphorylation process of Akt1 and protects Akt1 from dephosphorylation to maintain its activity. On the other hand, Hsp90 can combine with PDK-1 to enhance its stability, which increases the activation of Akt1 (Sato et al., 2000; Prodromou and Pearl, 2003; Giulino-Roth et al., 2017). So, we hypothesized that HAX-1 might facilitate the interaction of Hsp90 and Akt1 by binding to Hsp90. As demonstrated in the results of immunofluorescent co-location, HAX-1 knock-out decreased the co-location of Hsp90 and Akt1, compared with control and OE group. Interestingly, images of the co-location studies showed Akt1 and Hsp90 to be co-localized near the membrane-substrate interface when HAX-1 was expressed, in the region where it is known that Akt1 is phosphorylated by the PI3K/PDK1 axis (Zhao et al., 2017). The knock-out of HAX-1 destroyed the peripheral, ring-like structure of the co-localized Hsp90 and Akt1, which might lead the decline of the phosphorylation of Akt1. The Co-IP test also confirms the hypothesis that, compared with control group, the amount of Akt1 or Hsp90 baited by each other was significantly decreased in the HAX-1 knock-out group, while this trend was reversed by the overexpression of HAX-1. These results support our hypothesis that HAX-1 could regulate the phosphorylation of Akt1 by enhancing the binding of Akt1 and Hsp90.

Through these experiments, we demonstrated for the first time that HAX-1 could regulate cell proliferation and apoptosis of glioblastoma cells by affecting the activation of the Akt1 signal pathway via interaction with Hsp90. By down-regulating the expression of HAX-1, we were able to induce cell cycle arrest and apoptosis of glioblastoma cells as p53 expression increased.

## Author Contributions

X-bG designed the experiment. XD and YW performed the experiment. X-bG wrote the manuscript. All authors discussed and contributed to the analysis of the experimental data.

## Conflict of Interest Statement

The authors declare that the research was conducted in the absence of any commercial or financial relationships that could be construed as a potential conflict of interest.
