# Corrigendum: HAX-1 Protects Glioblastoma Cells From Apoptosis Through the Akt1 Pathway

**DOI:** 10.3389/fncel.2019.00013

**Published:** 2019-01-31

**Authors:** Xin Deng, Laijun Song, Wen Zhao, Ying Wei, Xin-bin Guo

**Affiliations:** ^1^Department of Neurosurgery, The First Affiliated Hospital of Zhengzhou University, Zhengzhou, China; ^2^Key Laboratory of Advanced Pharmaceutical Technology, Ministry of Education of China, Zhengzhou, China; ^3^Co-innovation Center of Henan Province for New Drug R & D and Preclinical Safety, Zhengzhou, China; ^4^School of Pharmaceutical Sciences, Zhengzhou University, Zhengzhou, China; ^5^Department of Neuro-interventional Radiology, The First Affiliated Hospital of Zhengzhou University, Zhengzhou, China

**Keywords:** glioblastoma, HAX-1, Akt1, Hsp90, apoptosis

In the original article, there was a mistake in Figure 1B U118 KO-1 and KO-2 as published. In Figure 1, the images for B U118 KO-1 and KO-2 were incorrectly provided. The corrected Figure 1B U118 KO-1 and KO-2 appears below.

**Figure 1:**
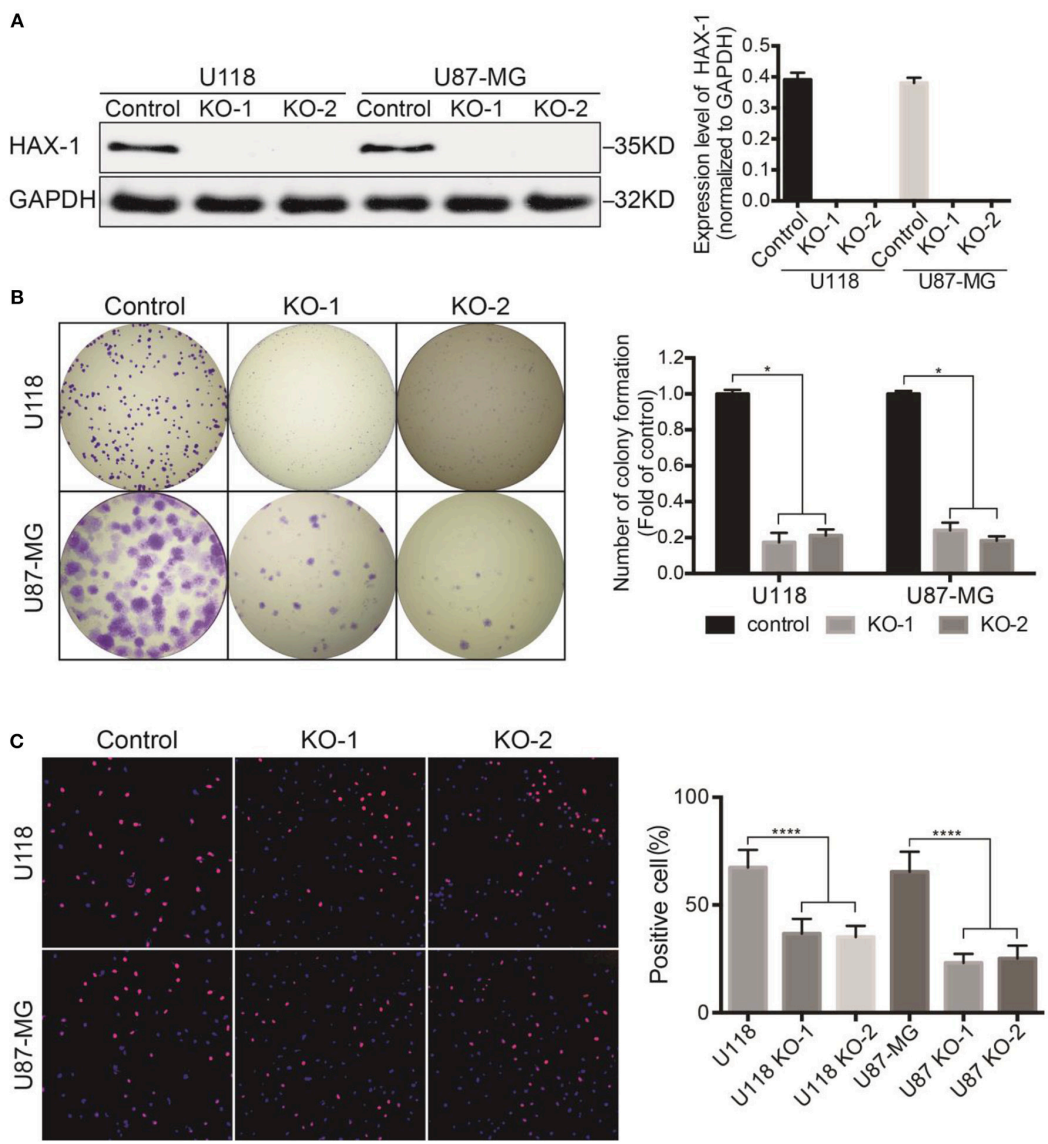
HAX-1 regulated cell proliferation of glioblastoma cells. **(A)** Western blot showed that HAX-1 was completely knocked out in U118 and U87-MG. GAPDH was used as a loading control. **(B)** Colony formation assays indicated that the efficiency of colony formation of U118 and U87-MG cells declined after HAX-1 was knocked out. **(C)** Edu proliferation assays showed decreased proliferative U118 and U87-MG cells. Edu was labeled with red fluorescence and nuclei were stained with blue fluorescence. (magnification: 100×) Three individual experiments were performed for each group. ^*^P < 0.05, ^****^P < 0.0001.

The authors apologize for this error and state that this does not change the scientific conclusions of the article in any way. The original article has been updated.

## Conflict of Interest Statement

The authors declare that the research was conducted in the absence of any commercial or financial relationships that could be construed as a potential conflict of interest.

